# Interactions among Lung Cancer Cells, Fibroblasts, and Macrophages in 3D Co-Cultures and the Impact on MMP-1 and VEGF Expression

**DOI:** 10.1371/journal.pone.0156268

**Published:** 2016-05-27

**Authors:** Xiao-qing Liu, Rosemarie Kiefl, Claudia Roskopf, Fei Tian, Rudolf M. Huber

**Affiliations:** 1 Division of Respiratory Medicine and Thoracic Oncology, Medizinische Klinik V, Ludwig-Maximilians University (LMU), Thoracic Oncology Centre Munich, Comprehensive Pneumology Center Munich, Member of the German Center for Lung Research (DZL), Munich, Bavaria, Germany; 2 Department of medical oncology, First people's hospital of Lianyungang city, Lianyungang, Jiangsu, China; 3 Division of Hematooncology, Medizinische Klinik-Innenstadt, Ludwig-Maximilians University (LMU), Munich, Bavaria, Germany; University of Nebraska Medical Center, UNITED STATES

## Abstract

*In vitro* cell-based models of lung cancer are frequently employed to study invasion and the mechanisms behind metastasis. However, these models often study only one cell type with two-dimensional (2D) monolayer cell cultures, which do not accurately reflect the complexity of inflammation *in vivo*. Here, a three-dimensional (3D) cell co-culture collagen gel model was employed, containing human lung adenocarcinoma cells (HCC), human lung fibroblast cells (MRC-5), and macrophages. Cell culture media and cell images were collected, and matrix metalloproteinase-1 (MMP-1) and vascular endothelial growth factor (VEGF) production was monitored under different cell culture conditions. We found that simulating hypoxia and/or serum starvation conditions induced elevated secretion of VEGF in the 3D co-culture model *in vitro*, but not MMP-1; the morphology of HCC in the 2D versus the 3D co-culture system was extremely different. MMP-1 and VEGF were secreted at higher levels in mixed cell groups rather than mono-culture groups. Therefore, incorporating lung cancer cells, fibroblasts, and macrophages may better reflect physiological metastasis mechanisms compared to mono-culture systems. Tumour stromal cells, macrophages, and fibroblast cells may promote invasion and metastasis, which also provides a new direction for the design of therapies targeted at destroying the stroma of tumor tissues.

## Introduction

Reports of pulmonary malignancies date back as far as antiquity, and by the mid-twentieth century, lung cancer had become epidemic and was firmly established as the leading cause of cancer-related deaths in North America and Europe, following the introduction of cheap, mass-produced cigarettes [1]. At present, lung cancer is the most common type of cancer worldwide, accounting for over 1.35 million cases per year [2]. Despite significant advances in the treatment of the early stages of disease, survival rates for advanced stages of lung cancer remain low; the majority of late stage lung cancer patients die within 18 months of diagnosis [3]. Accumulating evidence demonstrates that while most cancer researchers focus exclusively on lung cancer cells, there is a growing recognition that the tumor microenvironment (TME) and tumor-stromal interactions play important roles during the process of lung cancer establishment, invasion, and metastasis. The tumor stroma consists of both extracellular matrix (ECM) and cellular components. The ECM includes secreted proteoglycans that play both a structural and cell-signaling role. Cellular components include immune cells, cancer-associated fibroblasts (CAFs), endothelial cells, adipocytes, bone marrow-derived cells, myofibroblasts, and fibroblasts [4]. Functional genomic studies have identified gene signatures that are prognostic for non-small lung cancer (NSCLC) survival, including genes encoding ECM-proteins [5], which highlights the role of the stroma in patient prognosis and survival.

In this study, we chose to investigate MMP-1 and VEGF as indicators of the interaction between tumor and stromal cells, because both have been clearly linked to tumor invasion and metastasis. Previous studies demonstrate that MMPs play a salient role in cancer [6]. MMPs can degrade various proteins associated with the ECM. As a result, tumor cells may move more readily during the processes of invasion and metastasis. MMP-1 belongs to the MMP family and is known as collagenase or gelatinase, which degrades collagen IV and is increased in highly metastatic cancer cells [7]. VEGF was identified and isolated as an endothelial cell-specific mitogen, which has the capacity to induce physiological and pathological angiogenesis essential for establishing new blood vessels [8, 9]. In a solid tumor, during the expansion process, the central parts of the tumor become hypoxic, which promotes VEGF production and consequently, the proliferation of the tumor [10]. Additionally, recent studies have shown that VEGF-induced activities in tumor cells include tumor invasion and metastasis [11].

The majority of research groups have used cell monolayers grown on tissue culture plastics, which are less complex, less adaptable, and not very representative of the physiological extracellular microenvironment present in humans [12]. When cells are cultured on the stiff plastic surfaces of culture flasks, they only can grow away from the plastic in a 2-dimensional (2D) manner. In addition, many studies indicate that 2D cell cultures cannot reflect adequately the physiological complexity of real tissue, and their use in cell-based assays to some extent might result in errors in predicting tissue-specific responses. Cells cultured in 2D formats undergo proliferation and then de-differentiation, consequently losing their essential functions [13]. In contrast, cells cultured in a 3D matrix are dramatically different in terms of proliferation, differentiation, morphology, and cellular functionality [14, 15]. In this study, we established an organotypic co-culture model composed of lung adenocarcinoma cells (HCC), lung fibroblasts (MRC-5), and immune cells (macrophage), which not only enables the exploration of the interactions between tumor cells and stromal cells, but also represents a model that is more reflective of the conditions present *in vivo*.

## Materials and Methods

### Cell culture

All the cell lines were provided by the Medical Clinic V Laboratory of Ludwig-Maximilians University. MRC-5 cells were grown in Eagle’s Minimum Essential Medium (LGC Standards GmbH, Wesel, Germany). To make the complete growth medium, we added fetal bovine serum (FBS) at a final concentration of 10%. HCC cells were grown in RPMI 1640 supplemented with 10% FBS (Biochrom AG, Berlin, Germany). Macrophages were grown in Ham’s F-12 K medium (LGC Standards GmbH, Wesel, Germany) supplemented with 100 U/ml penicillin, 100 mg/ml streptomycin solution, and 2 mM L-Glutamine (PAA).

### 3D collagen gel co-culture model

Before preparation of the collagen gel, cells were thawed and diluted to 2 x 10^5^ cells per well. The ratio of cells was established as follows: 5:5:1 HCC: MRC-5: macrophage. For Western blot assays, cells were placed into 2 x 10^5^ and 1 x 10^6^ cells per group. To prepare the gels, collagen (Invitrogen, Darmstadt, Germany), sterile 10X phosphate buffered saline (PBS), sterile distilled water (ddH_2_O), and sterile 1N NaOH were mixed on ice. The total volume of collagen gel was calculated as follows:

$$Volume\;of\;collagen\;required\;\left(V1\right)=\frac{(Final\;conc.\;of\;collagen)\times \left[Total\;Volume\;\left(Vt\right)\right]}{Initial\;conc.\;of\;collagen}$$

$$Volume\;of\;10X\;PBS\;required\;\left(V2\right)=\frac{Total\;Volume\;\left(Vt\right)}{10}$$

Volume of 1N NaOH required (V3)=(V1)×0.025

$$Volume\;of\;dH_{2}O\;required\left(V4\right)=\left(Vt\right)-\left(V1+V2+V3\right)$$

In a sterile tube mix the dH_2_O, 1N NaOH, and 10X PBS.

The final concentration of collagen was 1 mg/ml, and 0.5 ml was dispensed into each culture dish compartment and immediately placed on ice. The cells were then seeded into the collagen gel, which was pipetted several times to mix well. The gels were removed to room temperature where they solidified rapidly. Cultures were then incubated at 37°C in a humidified incubator for 30–40 minutes, or until a firm gel was formed. Each dish compartment then received a total volume of 1.0 ml culture media.

### Western blotting

Cell culture supernatants were collected at 48 h. Vivaspin tubes (Sartorius Stedim Biotech GmbH, Goettingen, Germany) were used to collect proteins from the supernatant that had a higher molecular weight than 30 kDa. Protein concentrations were measured with a non-interfering protein assay kit (Calbiochem, EMD Bioscience Inc., Darmstadt, Germany) using a bovine serum albumin (BSA) standard curve (Bio-Rad Laboratories, Munich, Germany)

An anti-MMP-1 antibody produced in mice was used (Sigma-Aldrich Saint Louis, USA) with a goat anti-mouse secondary IgG-HRP antibody (Santa Cruz Biotechnology Inc., Heidelberg, Germany). 40 μg protein from each sample was diluted with sample buffer, while ddH_2_O was added to a final volume of 35 μl. The membranes were blocked in blocking buffer at room temperature for at least 1 h and afterward incubated with primary antibody overnight at 4°C. The primary antibodies were diluted 1:200 in blocking buffer. The membranes were incubated with diluted 1:5000 HRP-conjugated secondary antibodies for 1h at room temperature the following day. Images were analyzed using image reader LAS-R software (Leica Microsystems, Germany). IOD (integrated optical density) values were generated/analyzed with Gel-pro analyzer software (Media Cybernetics USA).

### HCC and Macrophage cells

Cells were washed twice using pre-warmed (37°C) PBS to remove any residual cell culture medium. We then added 10 ml pre-warmed (37°C) CFDA SE Cell Tracer working solution (Invitrogen, Darmstadt, Germany). The cells were then incubated for 15 minutes at 37°C. We then replaced the loading solution with fresh, pre-warmed medium and incubated the cultures for another 30 minutes at 37°C. This technique stained HCC, macrophage cells, and MRC-5 cells within the 3D collagen gel model described earlier. After 48 h of co-culture, the cell morphologies were observed by confocal microscopy.

### Frozen sections of collagen gels stained with Phallotoxins and DAPI

Frozen sections were prepared after 48 h of 3D collagen gel co-culture, and the specimens were mounted on coverslips at room temperature for 30 min. Samples were then washed three times with PBS for 3 min each time. Samples were then fixed in 3.7% formaldehyde in PBS for 10 minutes at room temperature. Samples were then washed three additional times with PBS. Each coverslip was then placed in a glass petri dish and extracted with a solution of 0.1% Triton X-100 in PBS for 3 to 5 minutes and washed again three times with PBS. When staining with the fluorescent phallotoxins (Invitrogen, Darmstadt, Germany), we diluted 5 μl methanolic stock solution into 200 μl PBS for each coverslip to be stained. To reduce nonspecific background staining with these conjugates, we added 1% BSA to the stain solution. Coverslips where then placed in staining solution for 20 minutes at room temperature. After staining, coverslips were washed three times with PBS. Counterstaining was performed with DAPI (Invitrogen, Darmstadt, Germany). DAPI stock solution was diluted to 300 nM in PBS, and approximately 300 μl of this DAPI staining solution was added to the coverslip preparation, completely covering the coverslips. Coverslips were incubated for 1–5 minutes and rinsed three times in PBS for 10 min. Samples were then imaged using a fluorescence microscope. The samples were air-dried, mounted, and stored in the dark at 2–6°C.

### Elisa

Cell culture supernatants were collected at different time points and stored at -20°C. A human MMP-1 ELISA Kit (Sigma-Aldrich Saint Louis, USA) and human VEGF DuoSet ELISA Kit (R&D Systems GmbH. Wiesbaden, Germany) were used to measure the levels of these proteins according to the manufacturer’s instructions.

### Statistics

All experiments were performed in triplicate for a minimum of three independent experiments. The SPSS 19.0 software (International Business Machines Corporation) package was used for statistical analysis. Data are depicted as the mean ± standard deviation. Differences between two groups were compared via a ***t-***test. When three or more groups were analyzed, one-way ANOVA and pearson correlations or SNK tests were used. *P* < 0.05 denotes a statistical difference.

## Results

### Expression of MMP-1 in 3D mono- or co-culture lung cancer models

HCC, MRC-5, and macrophage co-culture groups, along with MRC-5, HCC, and macrophage mono-culture groups were cultured in 10% FBS and O_2_, as described in the methods. Every group had 2 x10^5^ cells seeded, and the ratio of HCC, MRC-5, and macrophages in the co-culture group was 5:5:1, and the HCC and MRC-5 co-culture group was 1:1.

**Fig 1:**
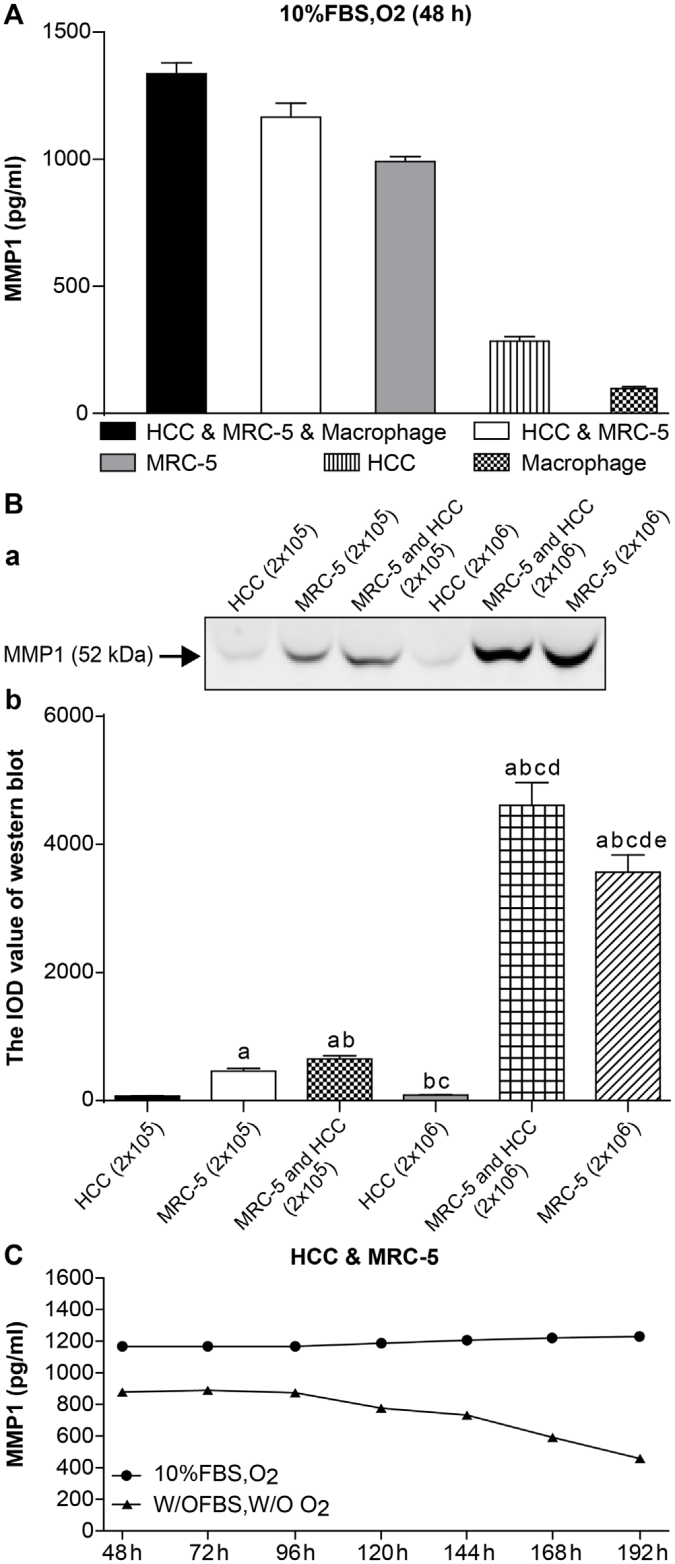
The expression of MMP1. **(A) Expression of MMP-1 in 3D mono- and co-culture lung cancer models at 48 h detected by ELISA.** The expression of MMP1 in HCC & MRC-5 & macrophage co-culture group was higher than that in HCC & MRC-5 co-culture group, or MRC-5/HCC/macrophage mono-culture groups. There was almost no expression of MMP1 in the HCC/macrophage mono-culture group. **(B) Expression of MMP-1 in 3D mono- and co-culture lung cancer model at 48 h detected by Western blotting.** In Fig 1B, a, the molecular weight of MMP-1 is 52 kD. From the left to right, the lanes are: HCC mono-culture group (2 x 10^5^ cells); MRC-5 mono-culture group (2 x 10^5^ cells); MRC-5 and HCC co-culture group (2 x10^5^ cells); HCC mono-culture group (1 x 10^6^ cells); MRC-5 and HCC co-culture group (1 x 10^6^ cells); MRC-5 mono-culture group (1 x 10^6^ cells). Expression of MMP-1 in co-culture groups was higher than in mono-culture groups (both 2 x 10^5^ cells and 1 x 10^6^ cells). Expression of MMP-1 in the 1 x 10^6^ cell group was higher than the 2 x 10^5^ cell group, regardless of mono-culture or co-culture group designations. In Fig 1B, b, the mean IOD values of the Western blot are shown. **(C) Expression of MMP-1 under different co-culture conditions.** Expression of MMP1 under 10% FBS and O_2_ (10% FBS cell culture medium with O_2_) was higher than that under w/o FBS and w/o O_2_ (without FBS and without O_2_) at 7 different time points. Furthermore, the expression trend of MMP1 under the condition of w/o FBS and w/o O_2_ continued to decline from 120 h.

After 48 h, the expression of MMP-1 in the HCC, MRC-5, and macrophage co-culture group (1337.00 ± 42.43) was higher than in the HCC and MRC-5 co-culture group (1166.25 ± 56.21), which was also higher than the MRC-5 mono-culture group (991.50 ± 19.09) and was significantly higher than the HCC (284.00 ± 18.38) and macrophage (98.50 ± 7.12) mono-culture groups. HCC and macrophage mono-culture groups exhibited almost no MMP-1 expression. MMP-1 was significantly higher in co-culture groups than mono-culture groups (n = 3, *P* < 0.05, Table 1 and Fig 1A, detected by ELISA).

**Table 1:** Statistical analysis of the expression of MMP-1 by ELISA assay (pg/ml).

	Mean	SD	F	*P*
HCC & MRC-5 & Macrophage	1337.00^a^^,^^b^^,^^c^^,^^d^	42.43	1062.983	<0.001
HCC & MRC-5	1166.25^a^^,^^b^^,^^c^	56.21		
MRC-5	991.50^a^^,^^b^	19.09		
HCC	284.00^a^	18.38		
Macrophage	98.50	7.12		

Compared with macrophage mono-culture group,

^**a**^ *P* < 0.05; with HCC-mono-culture group,

^**b**^ *P* <0.05; with MRC-5 mono-culture group,

^**c**^ *P* <0.05; with HCC & MRC-5 co-culture group,

^**d**^ *P* <0.05.

The expression of MMP-1 was further investigated by Western Blot. HCC and MRC-5 mono-culture groups and the HCC and MRC-5 co-culture groups were divided into 2 x 10^5^ cells and 1 x 10^6^ cell groups, as described in the methods. The ratio of the HCC and MRC-5 co-culture group was 1:1. We found that the expression of MMP-1 in co-culture groups was higher than in mono-culture groups, both in the 2 x 10^5^ cell group and 1 x 10^6^ cell groups. Moreover, the expression of MMP-1 in the 1 x 10^6^ cell groups was higher than the 2 x10^5^ cell groups, irrespective of mono-culture or co-culture grouping (n = 5, P < 0.05, Table 2 and Fig 1B).

**Table 2:** Statistical analysis of the expression of MMP-1 by Western Blot (IOD value).

	Mean(IOD)	SD	F	*P*
Lane 1	72.27	5.56	1155.896	< 0.001
Lane 2	464.92^a^	35.76		
Lane 3	651.21^a^^,^^b^	50.09		
Lane 4	87.53^b^^,^^c^	6.73		
Lane 5	4611.50^a^^,^^b^^,^^c^^,^^d^	354.73		
Lane 6	3565.40^a^^,^^b^^,^^c^^,^^d^^,^^e^	274.26		

Compared with Lane 1,

^**a**^ *P* < 0.05; with Lane 2,

^**b**^ *P* < 0.05; with Lane 3,

^**c**^ *P* < 0.05; with Lane 4,

^**d**^ *P* < 0.05; with Lane 5,

^**e**^ *P* < 0.05.

### The expression of MMP-1 in a 3D co-culture lung cancer model under different co-culture conditions

The expression of MMP-1 in HCC and MRC-5 co-culture model was analysed under different culture conditions: 10% FBS and O_2_ (10% FBS cell culture medium with O_2_), and with neither (without FBS and O_2_) to explore the effect of simulating hypoxia and starved of fetal bovine serum condition on MMP-1 secretion. Cell culture supernatants were collected separately from 3D co-culture collagen models at seven different time points from 48 to 192 h. Every group had an equal number of cells (2 x 10^5^) with a ratio of 1:1. We found that the expression of MMP-1with 10% FBS and O_2_ was higher than the expression without FBS and O_2_ for all seven time points. Furthermore, MMP-1 expression without FBS and O_2_ declined from 120–192 h (n = 3, P < 0.05, Table 3 and Fig 1C).

**Table 3:** Statistical analysis of the expression of MMP-1 under normal, hypoxic and serum starvation conditions (pg/ml).

	10% FBS, O_2_		w/o FBS, w/o O_2_		t	*p*
	Mean	SD	Mean	SD		
48 h	1166.50	87.73	883.00	67.07	5.740	< 0.001
72 h	1163.50	82.50	888.50	63.46	5.908	< 0.001
96 h	1161.00	89.31	871.00	61.21	5.989	< 0.001
120 h	1185.00	91.15	775.50	56.39	8.543	< 0.001
144 h	1208.50	95.96	733.50	52.39	9.715	< 0.001
168 h	1219.00	93.77	588.00	42.00	13.732	< 0.001
192 h	1226.50	94.35	454.50	32.46	17.301	< 0.001

### Expression of VEGF in 3D mono or co-culture lung cancer models

HCC, MRC-5, and macrophage co-culture groups, along with MRC-5, HCC, and macrophage mono-culture groups were cultured in 10% FBS and O_2_, as described in the methods. Every group had 2 x10^5^ cells seeded and the ratio of HCC, MRC-5, and macrophages; the co-culture group was 5:5:1 and the HCC and MRC-5 co-culture group was 1:1.

**Fig 2:**
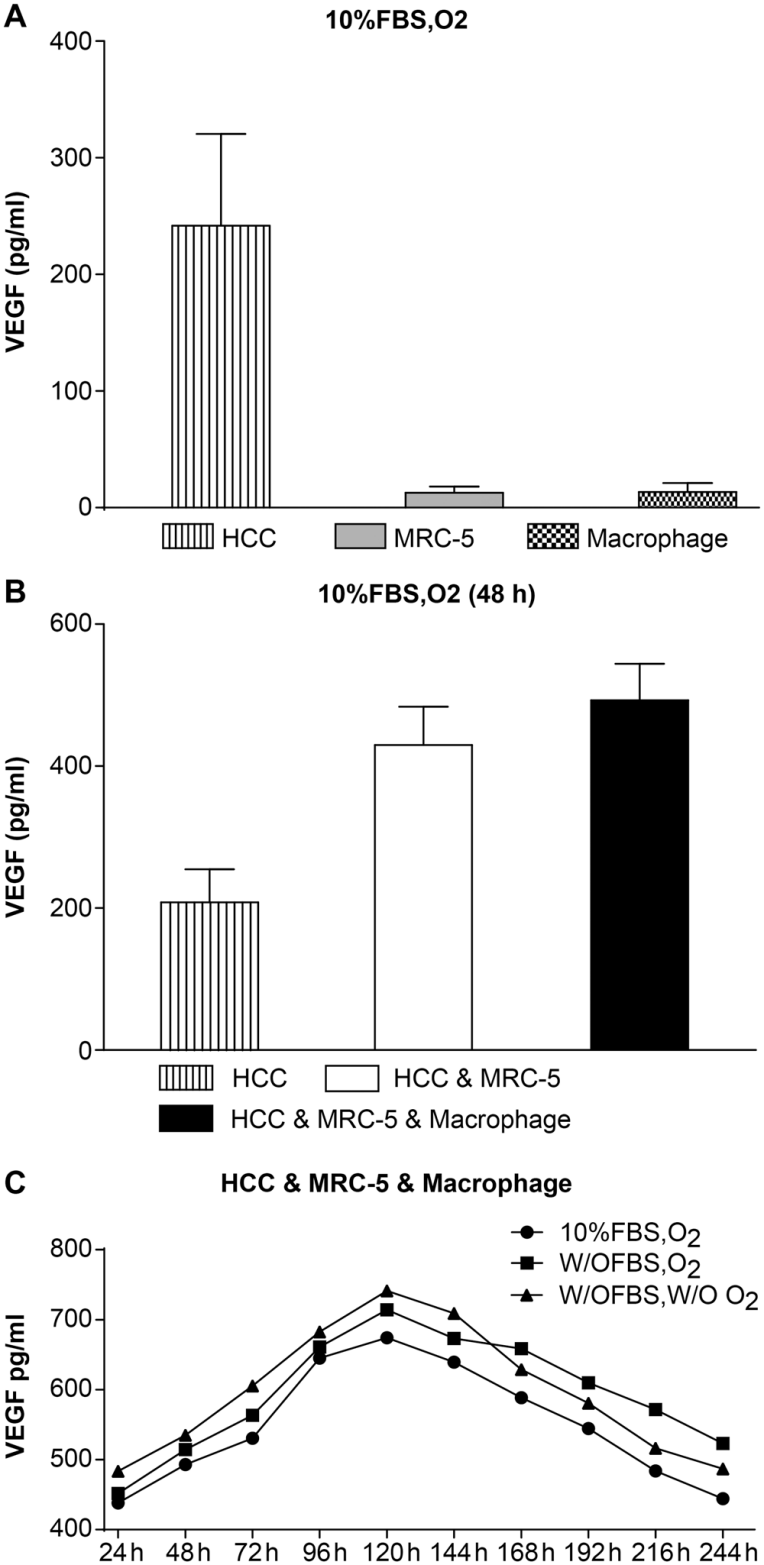
The expression of VEGF. **(A) Expression of VEGF in HCC, MRC-5, and macrophage mono-cultures groups.** Expression of VEGF in the HCC mono-culture group was significantly higher than expression in the MRC-5/macrophage mono-culture group under 10% FBS and O_2_ culture conditions. **(B) Expression of VEGF in HCC, MRC-5, and macrophage co-culture groups compared with the HCC mono-culture group.** Expression of VEGF in the HCC & MRC-5 & Macrophage co-culture group was higher than in the HCC & MRC-5 co-culture group and the HCC mono-culture group cultured with 10% FBS and O_2_ for 48 h. **(C) Expression of VEGF in HCC, MRC-5, and macrophage co-culture groups under different co-culture conditions.** The expression of VEGF in cells cultured w/o FBS (starved of FBS but with O_2_), w/o FBS and w/o O_2_ (without FBS and without O_2_) was higher than that in 10% FBS or O_2_ (10% FBS cell culture medium with O_2_), while the expression of VEGF in the three different conditions first increased and then decreased.

HCC, MRC-5, and macrophage mono-culture groups were cultured separately, and cell culture supernatants were collected after 48 h. We found that the expression of VEGF in the HCC mono-culture group (241.97 ± 78.56) was significantly higher than in the MRC-5 mono-culture (12.69 ± 5.46) and the macrophage mono-culture (13.65 ± 7.44) groups (n = 3, *P* < 0.05, Table 4 and Fig 2A).

**Table 4:** Statistical analysis of the expression of VEGF in HCC, MRC-5, and macrophage mono-culture groups (pg/ml).

	Mean	SD	F	*p*
HCC	241.97^a^^,^^b^	78.56	167.337	< 0.001
MRC-5	12.69^a^	5.46		
Macrophage	13.65	7.44		

Compared with macrophage mono-culture group,

^**a**^ *P* < 0.001; with MRC-5 mono-culture group,

^**b**^ *P* < 0.001.

The HCC, MRC-5, and macrophage co-culture group, HCC and MRC-5 co-culture group, and HCC mono-culture group (as control) were cultured separately, and cell culture supernatants were collected at 48 h. The expression of VEGF in both HCC, MRC-5, and macrophage (492.84 ± 51.43) and HCC and MRC-5 (429.63 ± 54.13) co-culture groups was higher than in the HCC mono-culture group (208.31 ± 46.45). The expression of VEGF in the HCC, MRC-5, and macrophage co-culture group was also higher than the HCC and MRC-5 co-culture group (n = 3, *P* < 0.05, Table 5 and Fig 2B).

**Table 5:** Statistical analysis of the expression of VEGF in HCC, MRC-5, and macrophage co-culture groups (pg/ml).

	Mean	SD	F	*P*
HCC & MRC-5 & macrophage	492.84^a^^,^^b^	51.43	51.951	< 0.001
HCC & MRC-5	429.63^a^	54.13		
HCC	208.31	46.45		

Compared with the HCC mono-culture group,

^**a**^ *P* < 0.05; with HCC & MRC-5 co-culture group,

^**b**^ *P* < 0.05.

### The expression of VEGF in the HCC, MRC-5, and macrophage 3D co-culture lung cancer model under different co-culture conditions

HCC, MRC-5, and macrophage co-culture groups were cultured under three different conditions: 10% FBS with O_2_ (10% FBS cell culture medium with O_2_), 0% FBS with O_2_ (starved of FBS but with O_2_), and without both (without FBS and without O_2_). Cell culture supernatants were collected at ten different time points. We found that the expression of VEGF under conditions with 0% FBS and O_2_ and without both was higher than the expression in the 10% FBS with O_2_ group, while the trend of VEGF expression in all the three conditions first increased, then decreased (n = 3, *P* < 0.05, Table 6 and Fig 2C).

**Table 6:** Statistical analysis of the expression of VEGF in HCC, MRC-5, and macrophage co-culture groups under three different co-culture conditions (pg/ml).

	10% FBS, O_2_		w/o FBS, O_2_		w/o FBS, w/o O_2_		F	*p*
24 h	438.24	38.71	451.54	34.73	483.40	47.18	3.277	0.053
48 h	492.84	51.43	514.42	29.57	534.81	31.14	2.944	0.070
72 h	530.72	31.59	563.26^a^	33.33	605.49^a^^,^^b^	36.58	12.232	< 0.001
96 h	645.12	20.39	661.01^a^	21.39	682.51^a^^,^^b^	25.50	6.934	0.004
120 h	674.24	32.63	714.24^a^	24.17	741.37^a^^,^^b^	27.03	14.378	< 0.001
144 h	639.22	39.17	673.26^a^	31.79	709.03^a^^,^^b^	34.54	9.780	0.001
168 h	588.58	26.04	658.70^a^	39.90	628.93^a^^,^^b^	29.15	11.910	< 0.001
192 h	544.53	31.89	609.77^a^	26.91	580.85^a^^,^^b^	25.45	13.420	< 0.001
216 h	483.90	37.22	571.46^a^	33.96	516.24^a^^,^^b^	32.63	16.321	< 0.001
244 h	444.11	34.16	523.22	40.25	486.97	37.46	3.501	0.063

Compared with 10% FBS and O_2_ condition group,

^**a**^ *P* < 0.05; with 0% FBS and O_2_ condition group,

^**b**^ *P* < 0.05.

### 2D and 3D co-culture model

**Fig 3:**
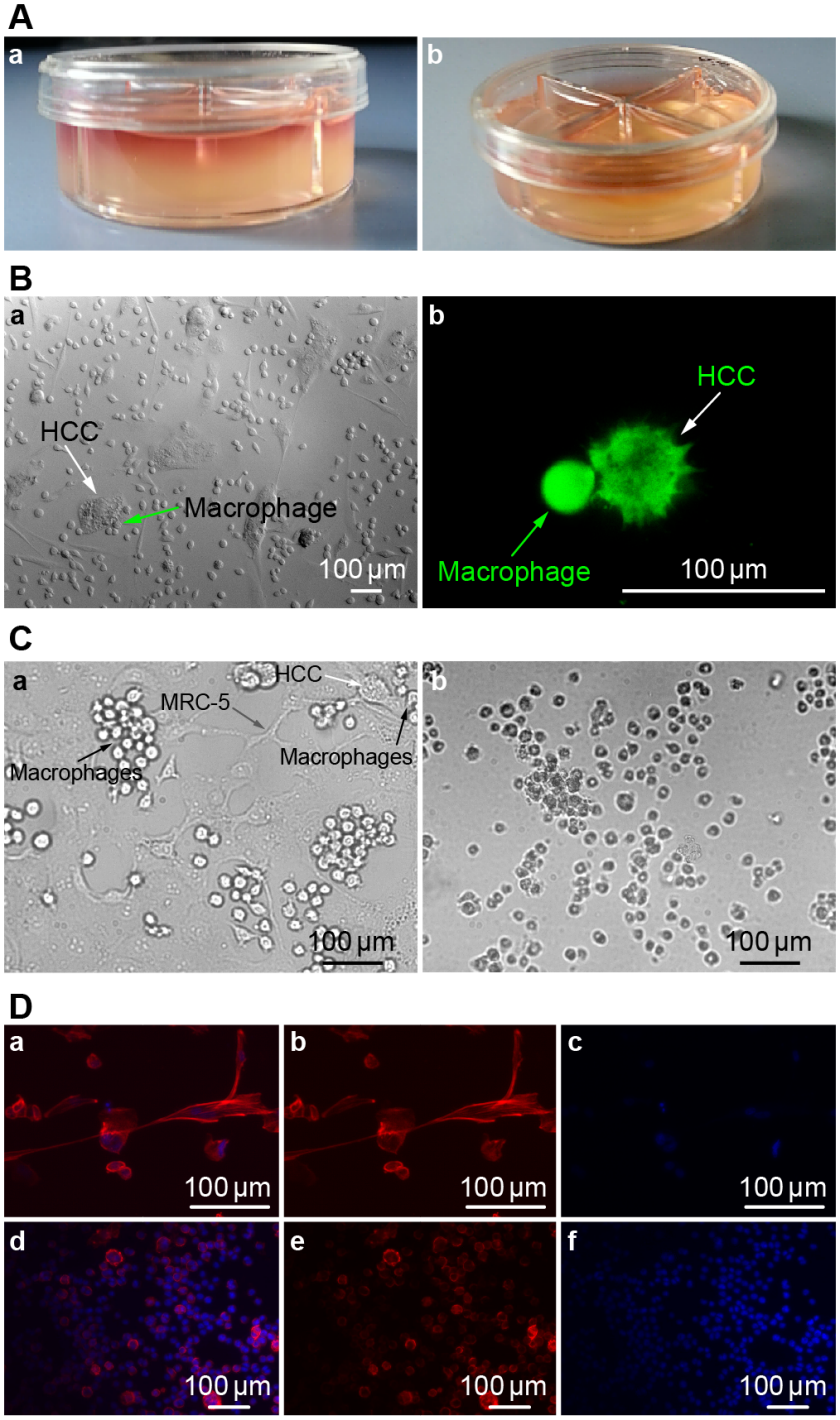
**(A) 3D collagen co-culture model.** Fig 3A,a: 1.0 ml cell culture medium permeated into the collagen gel from the top, and the cultivated cells were suspended in the 3D collagen gel space within. Fig 3A,b: 0.5ml collagen gel was firmed in every culture dish compartment independently. **(B) Cell morphologies in 2D and 3D co-culture models at 48 h.** Fig 3B,a: Image of HCC cells and macrophages in 2D co-culture model. Arrowhead denotes an HCC cell, which is flat and non-spherical. Arrowhead denotes macrophages surrounding an HCC cell at 40X magnification. Fig 3B,b: Image of HCC cells and macrophages in our 3D co-culture model. Arrowhead denotes an HCC cell, which is subglobose and prickly. Arrowhead denotes a macrophage. This image shows that these cells are in contact with one another, as viewed using CFDA SE Tracker on a confocal microscope. **(C) Images of HCC, MRC-5, and macrophage co-cultures at 48 and 168 h at 40X magnification.** Fig 3C,a: Macrophages contact and attack HCC cells after 48 h of co-culture. Arrowheads denote HCC, macrophage, and MRC-5 cells. Fig 3C,b: Co-culture after 7 days. As seen in this image after 7 days of culture, the cells began to detach and float in the medium, having lost their vitality and normal cell morphology. Most HCC and macrophage cells were dead. **(D) Fluorescence microscope images of MRC-5, macrophage, and HCC in 3D model.** Fig 3D (upper): Fluorescence microscope images of MRC-5, macrophage, and HCC cultures (Fig 3D,a). Cells were stained with DAPI (Fig 3D, c) and phallotoxins (Fig 3D, b). Fig 3D (lower): Fluorescence microscope images of Macrophages (Fig 3D,d). Cells were stained by DAPI (Fig 3D, f) and phallotoxins (Fig 3D, e).

A 3D collagen gel co-culture model was established (Fig 3A). Cell culture medium permeated into the collagen gel, and the cultivated cells were suspended in 3D space.

Cell morphologies in our 2D and 3D models were compared (Fig 3B). In the 2D co-culture model, the shape of the HCC cells was flat and non-spherical (Fig 3Ba), while in the 3D model, the HCC cells were subglobose and prickly (Fig 3Bb). Therefore, the shape of HCC cells was dramatically different between 2D and 3D models.

In addition, cell images collected after 48 h of co-culture showed that macrophages had contacted and attacked the HCC cells (Fig 3Ca), while after 7 days of co-culture we could see cells floating in the medium that lost their viability and had altered morphologies. Both the HCC and macrophage populations were largely dead (Fig 3Cb). Fluorescence microscope images of frozen sections of the MRC-5, macrophage, and HCC model and the 3D macrophage model were also collected (Fig 3D).

## Disscussion

The challenge in developing novel targeted therapies lies in establishing *in vitro* models that better reflect the conditions present *in vivo*. Early 2D models provided mechanistic insight into basic NSCLC metastasis. However, these model systems may not encompass the full range of signaling redundancy and/or compensatory mechanisms. 3D cell co-culture techniques that use cancer cells in combination with stromal cells could be a promising pathway to building a more representative model.

In our study, we established a three-dimensional co-culture collagen model with lung cancer adenocarcinoma cells, lung fibroblasts, and immune cells (macrophages). This model was devised to explore the tumor microenvironment and the interactions between lung cancer and stromal cells during the process of lung cancer invasion and metastasis. Roskelley et al. and Mitragotri et al. found that cells cultured in a 3D matrix showed dramatically different properties in terms of proliferation, differentiation, morphology, and cellular function [14, 15]. Similarly, we found that the morphology of HCC lung adenocarcinoma cells was significantly different between 2D and 3D co-culture models. In the 2D co-culture model, the shapes of the HCC cells were flattened and sheet-like, laying on the bottom of cell culture dish, while in the 3D model, the HCC cells were subglobose and prickly. This 3D co-culture lung cancer model not only provides an extracellular matrix for lung cancer cells, which is critical to their shape and function, but it also appears to better simulate the living environment and cellular interactions that occur *in vivo*.

In our study, we found that MMP-1 levels were almost undetectable in HCC and macrophage monoculture groups. VEGF levels were also almost undetectable in the MRC-5 and macrophages monoculture group, although both MMP-1 and VEGF were abundantly expressed in co-cultures of these cells. These findings demonstrate the interactions between lung cancer and stromal cells are important for expression of these markers of metastasis. Moreover, the expressions of both MMP-1 and VEGF were highest in the model that combined all three cell types (HCC, MRC-5, and macrophages), even higher than the levels in the dual culture system with HCC and MRC-5. Our findings also suggest that macrophages in 3D co-culture models not only increase the expression of MMP-1, but also improve the ability of lung cancer cells to promote angiogenesis. However, during the process of co-culture, we found that the macrophages also attacked and killed HCC cells. This observation implies that the function of macrophages in lung cancer may be a “double-edged sword,” where they exhibit both anti-tumor and tumor-promoting effects relevant to invasion, metastasis, and lung damage. Some reports have associated an abundance of tumor-associated macrophages (TAM) with a poor patient prognosis [16, 17]. Thomsen et al. reported that cigarette smoking causes an inflammatory reaction within the lungs that recruits inflammatory cells, consequently altering cytokine secretion in such a way that leads to a predisposition for lung cancer [18]. Shaked et al. demonstrated that bone marrow-derived cells, such as lymphocytes, macrophages, neutrophils, and mast cells (MCs) are often recruited to the lung in response to lung damage; along with fibroblasts, endothelial cells, and pericytes, immune cells appear to help condition the tumor microenvironment (TME) [19].

Tumor microenvironmental oxygen deficiency (hypoxia) also increases the malignant behavior of cancer cells, in part via the hypoxia-inducible factor (HIF) family of transcription factors. HIFs regulate the expression of EMT-genes, as well as promote angiogenesis, cell-proliferation, and tissue remodeling [20]. In the present study, we detected the expression of VEGF in the HCC, MRC-5, and macrophage co-culture group under three different conditions: 10% FBS with O_2_ (normal condition), 0% FBS with O_2_ (starved of fetal bovine serum but with O_2_), and 0% FBS without O2 (simulating hypoxia and serum starvation conditions). We found that the expression of VEGF under hypoxia or serum starvation was higher than under normal conditions after 72 h of co-culture, while the trend in VEGF expression showed an initial increase, followed by an apparent decrease. This may be due to HCC cell death over time mediated by the macrophages, which caused a latent decrease in VEGF expression. As also implied, in addition to hypoxia, starvation conditions may promote the expression of VEGF *in vitro*. However, in the HCC and MRC-5 co-culture group, which was used to compare the expression of MMP-1 under hypoxia or serum starvation with normal conditions, showed no significant differences in expression. We interpreted this to mean that neither hypoxia or serum starvation are factors that influence the expression of MMP-1. Furthermore, the expression of MMP-1 under these conditions continuously declined over the 120 h experiment. One explanation may be that the cells exhausted the necessary nutrients in the culture medium and/or low oxygen tension caused them to grow poorly and/or stopped cell division.

It is time to the reconsider the current 2D models used to test lung cancer therapeutics as the tumor microenvironment, presence of stromal cells, ECM components, and signaling molecules greatly influence the appearance, health, and activities of cancer cells. We propose that a better approach would be to engineer therapies that are also targeted against the tumor microenvironment and stromal cells, which are also important for metastasis and angiogenesis. Therapies that target only and work directly on lung cancer cells may not be as effective, because they fail to address key factors involved in tumor progression. Lung cancer cells are heterogeneous and they possess different histological properties, along with the fact that they are genetically unstable during progression of disease; all of these are major reasons for the failure of lung cancer treatments that focus on very specific targets on lung cancer cells. In addition, a high level of drug resistance usually accompanies therapies targeted directly to tumor cells. In summary, we see the advantages of therapies targeting the tumor microenvironment and stromal cells: 1) they do not target a specific aspect of highly variable tumor cells, 2) they target stromal cells, which possess a stable genetic background and inheritable stability, and 3) stromal cells are less prone to genetic mutations and drug resistance, yet they have an important impact on tumor progression and metastasis, such that inhibiting their function may effectively slow or halt tumor progression/spreading.

## Conclusions

The tumor microenvironment is composed of cancer cells, interstitial cells, cytokines, and chemokines. Generally, the interstitial cells, including fibroblasts, immune cells, endothelial cells, and immature cells are derived from bone marrow. Focusing on understanding how the interstitial cells function in the tumor microenvironment may lead to improvements in anti-cancer therapies. Our work has demonstrated that the morphology of HCC cells is significantly different between 2D and 3D co-culture models. The expression of MMP-1 and VEGF is upregulated in 3D co-culture models compared to monoculture models, which demonstrates that the interactions between lung cancer cells and stromal cells may have a significant role in enabling and promoting metastasis. In the 3D co-culture lung cancer model, simulated hypoxia and starvation conditions induced the secretion of VEGF, but not MMP-1. Macrophages in the tumor microenvironment may serve dual roles in attacking tumor cells, while also upregulating signaling pathways that aid in tumor invasion and metastasis.

## Outlook

Therapies targeting tumor stromal cells may represent a more effective approach to combating cancer. However, little is currently known about the interaction between stromal and tumor cells; additional research is necessary to better understand the complex tumor environment, specifically how TAMs function. Combined targeting of TAMs and lung cancer cells is one option to consider. However, there needs to be a greater understanding of the unique features of the lung cancer microenvironment. We propose that future studies focus on cancer-associated fibroblasts (CAFS), TAMs, the pre-metastatic niche, and ECM-alterations to seek out new targets for anti-cancer therapies. It is imperative that we gain a better appreciation of the interaction(s) that occur between tumor and stromal cells, if we wish to design more efficient and effective cancer therapies.
